# Disseminated Kaposi sarcoma following COVID-19 in a 61-year-old Albanian immunocompetent man: a case report and review of the literature

**DOI:** 10.1186/s40001-021-00620-9

**Published:** 2021-12-20

**Authors:** Giulia Gardini, Silvia Odolini, Giovanni Moioli, Dorothea Angela Papalia, Vittorio Ferrari, Alberto Matteelli, Silvio Caligaris

**Affiliations:** 1 ASST Spedali Civili Hospital, University Division of Infectious and Tropical Diseases, Brescia, Italy; 2 University of Brescia, Brescia, Italy; 3 Division of Oncology, ASST Spedali Civili Hospital, Brescia, Italy

**Keywords:** HHV-8, Kaposi sarcoma, COVID-19, Latent infection, Reactivation

## Abstract

**Background:**

COVID-19 and its related anti-inflammatory treatment (steroids, immunomodulators) may induce the reactivation of latent bacterial, parasitic, and viral infections. According to our knowledge, no case of disseminated HHV-8-related Kaposi sarcoma (KS) after COVID-19 and its treatment has been described so far. Only one case of cutaneous KS concurrently with COVID-19 has been previously reported.

**Case presentation:**

We describe a case of disseminated KS in a 61-year-old immunocompetent Albanian man after hospitalization for COVID-19.

**Methods for literature research:**

We used PubMed as biomedical database for the literature research. We selected keyword combinations including “Kaposi sarcoma,” “HHV-8,” “immunocompetent,” “COVID-19,” “SARS-CoV-2,” and “steroids.” No time or language limitation was set. Titles and abstracts of selected articles were systematically screened. Articles were included in the examination if they were published under free access through the digital library of the University of Brescia (Italy), and provided full text. Articles were excluded if the topic was beyond the aim of our study. Finally, we selected 15 articles.

**Results:**

We describe a case of KS in COVID-19 patient and postulate that Interleukin-6 (IL-6) activity and steroid-induced immunodeficiency may play a major role in KS emergence. No published case of disseminated KS following COVID-19 in otherwise healthy individuals was found through the systematic literature review, despite the high incidence of COVID-19 in areas with medium–high prevalence of HHV-8 infection. This observation might be explained by the role of individual genetic susceptibility factors.

**Conclusions:**

SARS-CoV-2 infection and its treatment may lead to reactivation of several latent infections, including HHV-8 and its related clinical syndrome, Kaposi sarcoma.

## Background

COVID-19 and its related anti-inflammatory treatment (steroids, immunomodulators) can induce the reactivation of several latent infections, e.g., strongyloidiasis, hepatitis B and herpetic infections [1, 2]. Among herpetic viruses, reactivation of HHV-1, VZV, HHV-6, HHV-7, and EBV have been reported [3–5].

According to our knowledge, only one case of cutaneous HHV-8-related KS after COVID-19 has been described so far [6]. A woman with previous history of KS experienced new skin relapse during COVID-19 (histological confirmation). No information about the immune status of the patient, the clinical severity of COVID-19, and concomitant drugs were reported by authors.

KS is a tumor of lymphatic and vascular endothelial cells, of which 4 types are distinguished based on risk groups, clinical presentation, and prognosis [7]. HHV-8 (re)active infection is a fundamental element to develop the sarcoma, but normally host factors contribute to the pathogenesis, in particular, genetic susceptibility (e.g., in African or endemic KS) and immune status (e.g., AIDS-related KS) [8]. The visceral involvement is typically observed in immunosuppressed individuals (e.g., AIDS, solid organ transplant recipients) and African children. On the contrary, immunocompetent subjects usually experience localized cutaneous lesions.

We here describe a case of disseminated KS in an immunocompetent 61-year-old man after hospitalization for COVID-19.

## Case presentation

On February 9, 2021, a 61-year-old Albanian man was admitted to our division due to SARS-CoV-2 pneumonia. Co-morbidities were diabetes and arterial hypertension, and co-medications were metformin, bisoprolol, and ACE inhibitor. After a few days, he was transferred to Intensive Care Unit (ICU) for respiratory failure. He was put on invasive ventilation and remained intubated for 4 days. In the meantime, he was treated with remdesivir, high-dose steroids (minimum dosage was iv dexamethasone 6 mg daily; maximum dosage was iv dexamethasone 20 mg daily), and antibiotics (ceftriaxone, azithromycin). Blood exams showed transient viral-induced lymphopenia for 10 days (minimum value 540 cells/μL). HIV test resulted negative. On February 26, 2021, he was transferred to another clinic for neuromotor rehabilitation. Co-medications were the same as before hospitalization, except for subcutaneous enoxaparin due to jugular thrombosis in the site of previous central venous catheterization. Steroid therapy was administered for a total of 10 days. No signs or symptoms suspected of other infections were present at that time.

At the beginning of March, still in the rehabilitation facility, the patient experienced high fever and abdominal pain and the laboratory results showed anemia, thrombocytopenia, and high inflammatory markers (white blood cells 15,000 cells/μL, C-reactive protein 111 mg/L with normal value  < 5 mg/L). Piperacillin/tazobactam and levofloxacin were started empirically, while enoxaparin was suspended. After a week, the clinical condition of the patient further deteriorated; thereby, the antibiotic therapy was escalated (meropenem  +  linezolid). The absence of improvement and the limited diagnostic resources available in the rehabilitation structure led him back to our division at the beginning of April. The following conditions were considered as differential diagnosis: uncontrolled sepsis, reactivation of a latent infection (tuberculosis? cytomegalovirosis?), and a de-novo malignant hematological disease. Biochemistry revealed anemia of unclear etiology (no intestinal bleeding, no vitamin or iron deficiency, no hemolysis, no bone marrow dysfunction), increased inflammatory indices, some unspecific alterations in the peripheral smear (thrombocytopenia plus macro-platelets; inflammatory aspect of neutrophils and lymphocytes), and hypergammaglobulinemia (43%—normal range 11–18%). LDH and thyroid function were normal. Peripheral lymphocytic typing presented no clonality. Microbiological researches resulted negative (blood cultures, sputum for bacteria, fungi, and alcohol–acid-resistant bacilli, plasmatic EBV-DNA and Parvovirus B19-DNA, serology for *Leishmania*, *T. gondii* and *Bartonella*, IGRA test, serum *Aspergillus* antigen, fecal *H. pylori* antigen, HBsAg, HCV antibodies) except from a transient and low-level positivity of plasmatic CMV-DNA (658 copies/mL). HIV test was not repeated in this occasion. Autoimmune panel was negative. Neck ultrasound showed multiple non-colliquate and partly confluent adenopathies (max. 21 mm). Core needle biopsy of a cervical lymphadenopathy was executed. At the same time a computed tomography (CT) scan of the abdomen was performed with radiological signs of cholecystitis and splenomegaly (about 17 cm). Minor alterations were a mild pleural effusion, ascending colon coprostasis with proximal dilatation, and abdominal and inguinal lymph adenomegalies. Antibiotic treatment (piperacillin/tazobactam, later switched to ertapenem) was resumed and the patient received blood transfusion. After a week we obtained stable apyrexia, a stable hemoglobin level (from 7.8 to 9.5 mg/dL), increasing platelets count (from 37,000 to more than 1,00,000), and normalization of inflammatory markers. He was discharged home with the diagnosis of cholecystitis and parainfectious cytopenia, while awaiting the histological and microbiological results of the lymph node biopsy.

**Fig. 1:**
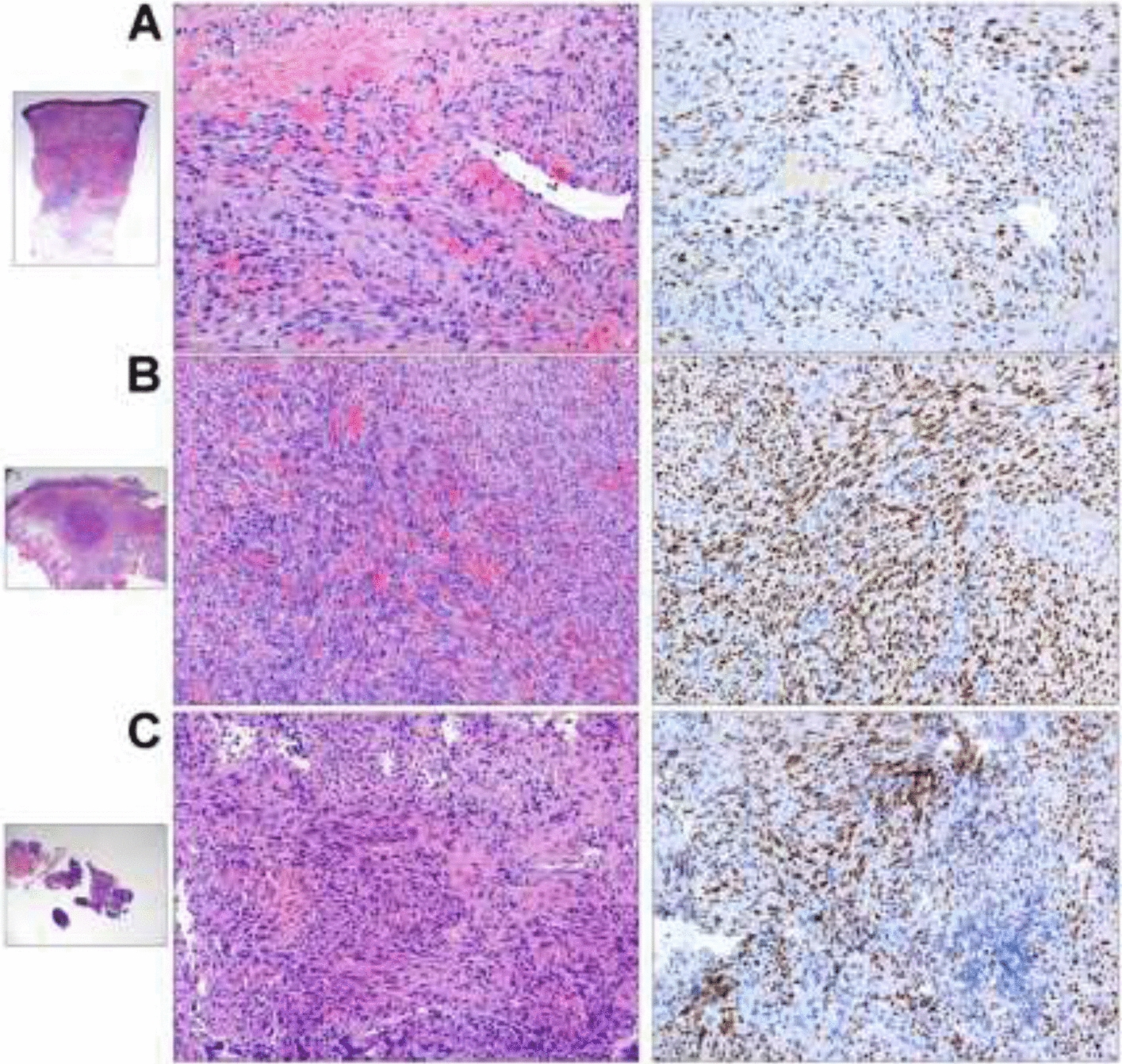
Histological evidence of HHV-8-related Kaposi sarcoma on patient’s skin, oral mucosa, and cervical lymph node. Courtesy of Prof. Fabio Facchetti, University Anatomic Pathology Division, Spedali Civili Hospital, Brescia (Italy). On the left, low power view of skin (**A**), oral mucosa (**B**), and cervical lymph node (**C**) biopsies, involved by Kaposi sarcoma. Images in the middle show abnormal vessels and spindle cell proliferation, with intermingled erythrocytes and figures on the right show the intense positivity of tumor cells for HHV-8 LANA-1 antigen (Left and middle figures: hematoxylin–eosin stain; right figures: immunohistochemistry using monoclonal antibody anti-LANA-1 HHV8-associated antigen, developed with diaminobenzidine and counterstained with Meyer’s hematoxylin)

**Fig. 2:**
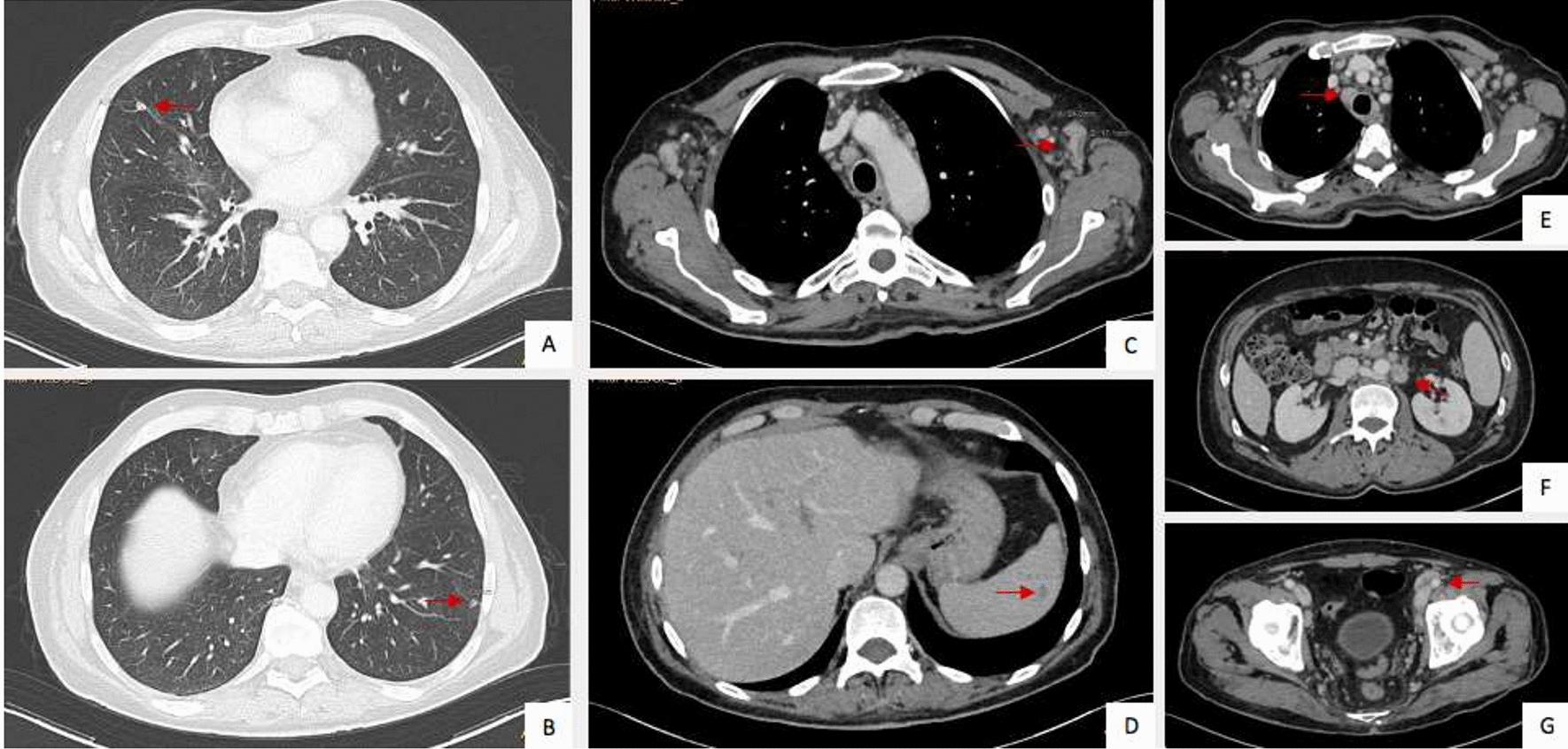
Disseminated Kaposi sarcoma revealed by the total-body CT scan. Courtesy of University Radiology Division, Spedali Civili Hospital, Brescia (Italy). CT scan images show lung parenchymal nodules (**A**, **B**), axillary lymph adenomegalies (**C**), splenic lesion (**D**), pre-tracheal (**E**), retroperitoneal (**F**), and inguinal (**G**) lymph adenomegalies

In May, we received an unexpected histological diagnosis of Kaposi sarcoma on the lymph nodal core needle biopsy previously performed (details in Fig. 1) and he was re-admitted to our ward. At physical examination he presented purple skin lesions on his forearms, palms, legs, and palate, that had appeared a few days before. Over the next 24 h, he developed further comparable skin lesions on the thorax. Histological diagnosis was re-confirmed on skin and palate biopsies (details in Fig. 1). Endoscopy of the gastrointestinal tract did not identify suspected lesions. Plasmatic HHV-8 DNA was 2306 copies/mL, and the molecular detection on tissue was not performed. No active infection of CMV, EBV, HSV 1–2, and HHV-6 was detected. IgG and IgM for HTLV-1 resulted negative. He remained negative for HCV, HBV, and HIV infections. The full-body CT scan revealed a disseminated disease (lung parenchymal nodules, liver and spleen lesions, deep lymph adenomegalies above and below the diaphragm, inguinal and axillary lymph nodes) (see Fig. 2). Chemotherapy was started.

After three cycles of well-tolerated liposomal doxorubicin, we obtained a partial improvement of skin and mucosal lesions, which appeared decreased in size and thickness.

## Methods for literature research

We used PubMed as biomedical database and selected keyword combinations including “Kaposi sarcoma,” “HHV-8,” “immunocompetent,” “COVID-19,” “SARS-CoV-2,” and “steroids.” No time or language limitation was set. Titles and abstracts of selected articles were systematically screened. Articles were included in the examination if they were published under free access through the digital library of the University of Brescia (Italy), and provided full text. Articles were excluded if the topic was beyond the aim of our study. Finally, we selected 15 articles.

## Discussion

Disseminated KS is frequently seen in HIV population with low CD4 +  cells count or HIV-uninfected patients with other immunosuppressive disorders as transplant recipients.

Our patient had no history nor immunological constellation of immune deficiencies. The only events that may have contributed to reactivate a latent HHV-8 infection were the recent COVID-19 and the administration of high-dose steroids. We conducted a literary search to determine if other similar cases have been reported.

At first, we searched for cases of disseminated KS in immunocompetent individuals (keyword combinations, including “sarcoma Kaposi” and “immunocompetent” and “HHV-8”) and we found only 1 case that had occurred in a 62-year-old man with a history of chronic alcoholism, diabetes, hypertension, and previous smoking [9]. The other results included 3 patients with HHV-8-related hemophagocytic syndrome (HS), but no evidence of KS, in a 61-year-old Taiwanese immunocompetent man [10] and two Italians over sixty years old following steroidal treatment for autoimmune hemolytic anemia [11]. A localized cutaneous KS, started as inguinal lymph node involvement, was reported in a 67-year-old Tunisian hypertensive and diabetic man [12]. 8 cases of cutaneous and/or mucosal KS in immunocompetent individuals were also reported (a 77-year-old Irish man, 2 men under 50 years old of unknown origin, a 55-year-old Afro-American man, 4 Turkish men of whom 3 over 70 years old and 1 of 27 years old) [13–16].

**Fig. 3:**
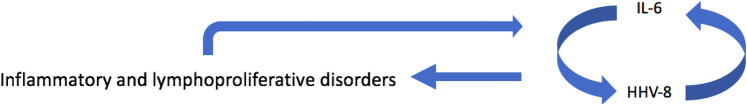
Connection between IL-6, HHV-8, and inflammatory/lymphoproliferative disorders

Some of the mentioned articles [8, 9] pointed to the role of IL-6 (Interleukin-6) in the pathogenesis of HHV-8-associated diseases (see Fig. 3). IL-6 production is stimulated by systemic viral infection (e.g., HHV-8, HIV), autoimmune diseases, hematologic disorders of lymphocytes, KS, and even by itself through a paracrine process. It then promotes inflammation and B cell proliferation, favoring B cell malignancies.

IL-6 biological activities and the positive effect of SARS-CoV-2 on its production [17] made us speculate that the underlying event implicated in the development of disseminated KS in our patient could be COVID-19. Leoni et al. [6] also speculated a similar role of IL-6 for their patient. A woman, with past history of KS and hospitalized for COVID-19, presented a concurrent relapse of skin KS. In skin biopsies both viral families (HHV-8, SARS-CoV-2) were detected by transmission electron microscope. Observing the reactivation of several herpesviruses, but not HHV-8, during COVID-19 [3–5], Chen et al. [18] performed an experimental laboratory study to test the effect of SARS-CoV-2 proteins and some anti-COVID-19 drugs (azithromycin, chloroquine, hydroxychloroquine, remdesivir, nafamostat mesylate) on reactivation of HHV-8. They revealed the capacity of both virus and azithromycin to manipulate intracellular signaling pathways toward reactivation of HHV-8 and thereby to favor HHV-8-related diseases as KS.

Steroidal therapy might also have played a role. Using key words combinations, including “steroids” and “Kaposi sarcoma,” we found only a few cases of KS induced by steroids [19–24]. Most of them occurred after long-term use of steroids prescribed for autoimmune or lymphoproliferative disorders; the disease was limited to the skin and partially regressed after steroids reduction or suspension. Visceral involvement was reported in 3 cases (1 case of stomach involvement after administration of steroids for osteoarticular pain, 1 case of intestinal KS in a 21-year-old Ethiopian male who has taken steroids for IgA nephropathy and with a recent diagnosis of Crohn’s disease, 1 case of disseminated KS in a 76-year-old woman with seronegative polyarthritis and idiopathic CD4 lymphocytopenia) [19, 20, 24].

In the light of the reported evidence, our case appears exceptional for several reasons. At first, disseminated KS is extremely rare in immunocompetent individuals. Steroids may induce KS, but generally if they are administered for prolonged period (not a few days as for our patient). Moreover, steroids-induced KS is usually a localized cutaneous or mucosal type. Considering the high incidence of COVID-19 in Italy and the comparable prevalence of latent HHV-8 infection among Italian and Albanian populations [25, 26], we cannot explain why no other cases of KS after COVID-19 have been reported until now. A possible explanation is that our patient may present concomitantly genetic susceptibility factors to develop HHV-8-related disease (not investigated). Further evidences and studies are needed.

## Conclusions

Reactivation of latent infections, including HHV-8, should be always considered when evaluating a patient with signs or symptoms of underlying infection and a recent history of COVID-19 and anti-COVID-19 steroidal therapy.

## Data Availability

Not applicable.

## Abbreviations

AIDS: Acquired immune deficiency syndrome
ACE: Angiotensin-converting enzyme
IGRA: Interferon gamma release assay
KS: Kaposi sarcoma
LDH: Lactate dehydrogenase
